# Hematogenous metastasis to colon from gallbladder cancer

**DOI:** 10.1002/jgh3.12542

**Published:** 2021-04-07

**Authors:** Daiki Murata, Atsushi Yamaguchi, Yuzuru Tamaru, Ryusaku Kusunoki, Toshio Kuwai, Hirotaka Kouno, Akira Ishikawa, Kazuya Kuraoka, Hiroshi Kohno

**Affiliations:** ^1^ Department of Gastroenterology National Hospital Organization Kure Medical Center and Chugoku Cancer Center Kure Japan; ^2^ Department of Pathology National Hospital Organization Kure Medical Center and Chugoku Cancer Center Kure Japan

**Keywords:** caudal‐type homeobox2, colon metastasis, cytokeratin 7, gallbladder cancer, hematogenous metastasis

## Abstract

Hematogenous metastasis to colon from gallbladder cancer is in rare situation and immunohistochemical staining is effective for differential diagnosis of the primary site of cancer.

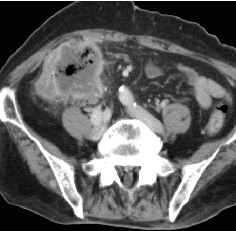

## Brief report

**Figure 1:**
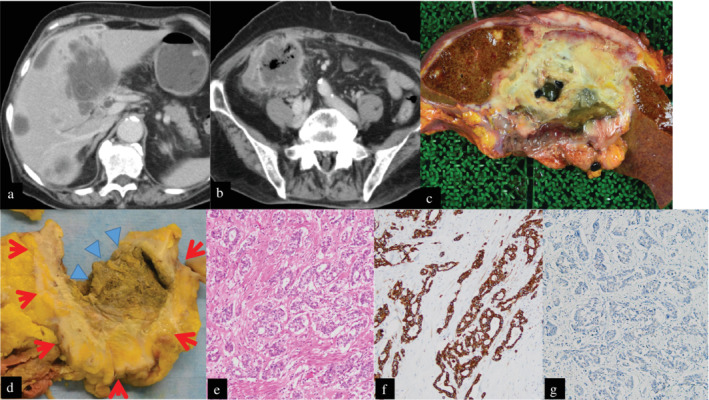
Contrast‐enhanced computed tomography scan of the abdomen showed a hypodense round mass around the bottom surface of the right liver lobe (a) and an ileocecal tumor (b). Her pathologic autopsy showed a 9‐cm tumor including gallstones in a typical gallbladder position (c) and a 5‐cm tumor with ulceration (arrowhead) in the wall (arrow) of the ascending colon and at the ileum end (d). The ileocecal tumor revealed poorly differentiated adenocarcinoma (e; HE staining, ×400). Immunohistochemical staining was positive for CK7 (f; ×100) and negative for CDX2 (g; ×400).

An 85‐year‐old woman was admitted to our hospital with a complaint of anorexia. Contrast‐enhanced computed tomography scan (CT) of the abdomen showed a hypodense round mass of 10 cm around the bottom surface of the right liver lobe and the mass had a connection with the duodenum (Fig. 1a). Furthermore, there were multiple liver hypodense lesions (Fig. 1a) and an ileocecal tumor of 7 cm with a similar shape to the abovementioned 10‐cm tumor (Fig. 1b). We considered this condition as liver metastasis from colon cancer or double cancer of the gallbladder and colon. Biopsy species from the duodenum using endoscopy showed poorly differentiated adenocarcinoma. The patient was in poor general condition because of a biliary infection, gastrointestinal bleeding, and poor nutrition and died before having histological species taken from the colon tumor. We performed her pathologic autopsy.

In a typical gallbladder position, a 9‐cm tumor with poorly differentiated adenocarcinoma was located including gallstones (Fig. 1c). Furthermore, a 5‐cm tumor with ulceration had grown in the wall of the ascending colon and at the ileum end. The tumor was located in all layers of the ascending colon wall and extend to the lumen side (Fig. 1d). The tumor showed poorly differentiated adenocarcinoma (Fig. 1e). The tumor cells were with short columnar epitheliums and they were atypical for colorectal carcinoma. Immunohistochemically, the tumors in the gallbladder and ascending colon were strongly positive for CK7 (Fig. 1f), CK20, and negative for CDX2 (Fig. 1g). The diagnosis was a primary gallbladder carcinoma and ileocecal metastasis.

Gallbladder cancers infiltrate to other organs with direct invasion, peritoneal dissemination, and metastasize hematogenously. Hematogenous metastasis of gallbladder cancer usually occurs in the liver, lung, and distinct lymph nodes and rarely in the colon. Edoardo et al. showed that the percentage of hematogenous metastasis to the colon from gallbladder cancer was only 2.8% in their 257 autopsy cases.^1^ In a search on PubMed, there were only two case reports of hematogenous metastasis to the colon from gallbladder cancer.^2^, ^3^ Our case was presumed to be hematogenous metastasis to the colon because the tumor was located mainly at the lumen side and there were no nodules suspected of peritoneal dissemination near the tumor.

In addition, a large colon tumor as a metastatic lesion was unusual. The abundant blood flow in the ileocecal submucosa might have promoted an increase in tumor cells.

If there are two or more tumors in the abdomen, it is necessary to distinguish which is the primary tumor or whether they are a double cancer. Immunohistochemical staining was reported to be effective for differential diagnosis of the primary site of cancer. Coordinate expression of CK7 and CK20 has also been proposed to help in determining the primary site of metastatic carcinoma.^4^, ^5^, ^6^ Duval et al.^7^ reported that pancreas, gallbladder, and bile duct carcinomas typically express a positive CK7 and negative CK20. In comparison, Kalekou et al.^8^ described that positivity for both CK7 and CK20 were found in moderate and poorly differentiated adenocarcinoma of the gallbladder 66.7% of the time. Therefore, the immunohistochemical profile of positive CK7 and positive CK 20 in our case was not a contradiction for diagnosis of gallbladder carcinoma. That said, differentiation with colon cancer is difficult. Gastric and colorectal carcinomas usually express negative CK7 and positive CK20,^5^, ^6^, ^7^ but Bayrak et al. reported that some colorectal carcinomas showed reactivity to CK7 (17.3%).^9^ In our situation, we diagnosed this case as gallbladder cancer due to a negative CDX2. Intestinal cancers (duodenum, small intestine, and large intestine) usually show a positive stain for CDX2. Werling reported that the expression of CDX2 was 99% in colonic adenocarcinoma, 70% in gastric adenocarcinoma, and 25% in cholangiocarcinoma plus gallbladder carcinoma.^10^ These characteristics also support the diagnosis of gallbladder cancer for our patient.

Initially, we diagnosed the tumors as metastatic liver tumors from colon cancer using CT, but the final diagnosis was colon metastasis from gallbladder cancer. It is important to carefully arrive at a diagnosis with the use of additional immunohistochemical examinations when there are similar tumors in multiple organs.

## Consent for publication

Written informed consent was obtained from the patient for the publication of this case report and accompanying images.
